# MicroAgroBiome: a toolkit for exploring specialized metabolism and ecological interactions in rhizosphere microbiomes of cultivated crops

**DOI:** 10.1093/nar/gkaf1083

**Published:** 2025-11-17

**Authors:** César Aguilar, Fernando Fontove-Herrera, Anton Pashkov, David A García-Estrada, Haydeé Contreras-Peruyero, Shaday Guerrero-Flores, Obed Ramírez-Sánchez, Nelly Sélem-Mojica

**Affiliations:** Industrial Genomics Laboratory, Centro de Biotecnología FEMSA, Escuela de Ingeniería y Ciencias, Tecnológico de Monterrey, Av. Eugenio Garza Sada 2501 Sur, Nuevo León C.P. 64700, México; C3 Consensus, León, Guanajuato, 37266, México; Escuela Nacional de Estudios Superiores, Unidad Morelia, Antigua Carretera a Pátzcuaro 8701 Col. Ex Hacienda de San José de la Huerta, C.P. 58089 Morelia, Michoacán, México; Departamento de Medio Ambiente y Energía, Centro de Investigación en Materiales Avanzados, Miguel de Cervantes 120, Complejo Industrial Chihuahua, Chihuahua C.P. 31136, México; Centro de Ciencias Matemáticas, Universidad Nacional Autónoma de México, Antigua Carretera a Pátzcuaro 8701, Col. Ex Hacienda de San José de la Huerta, C.P. 58089 Morelia, Michoacán, México; Centro de Ciencias Matemáticas, Universidad Nacional Autónoma de México, Antigua Carretera a Pátzcuaro 8701, Col. Ex Hacienda de San José de la Huerta, C.P. 58089 Morelia, Michoacán, México; Soil Genomics & Discovery Department, Solena Inc., Av. Olímpica 3020-D, Villas de San Juan, León 37295, México; Centro de Ciencias Matemáticas, Universidad Nacional Autónoma de México, Antigua Carretera a Pátzcuaro 8701, Col. Ex Hacienda de San José de la Huerta, C.P. 58089 Morelia, Michoacán, México

## Abstract

The microbiome is crucial to agroecosystems, as it influences plant nutrition, resilience, and overall health. Recent advances in metagenomics have expanded our understanding of plant–microbe interactions, yet curated, high-resolution data capturing the global diversity of crop-associated microbiomes remain scarce. To fill this gap, we developed MicroAgroBiome, a publicly accessible platform that offers standardized taxonomic and functional data, mainly from the rhizosphere microbiomes of agriculturally important crops. The platform integrates 554 metagenomes from 28 crops and soil sample health, advancing microbiome-informed agricultural strategies. It also underscores Latin America’s growing leadership in agricultural microbiome research. MicroAgroBiome is available at https://agrobiom.matmor.unam.mx.

## Graphical Abstract

**Graphical Abstract:**
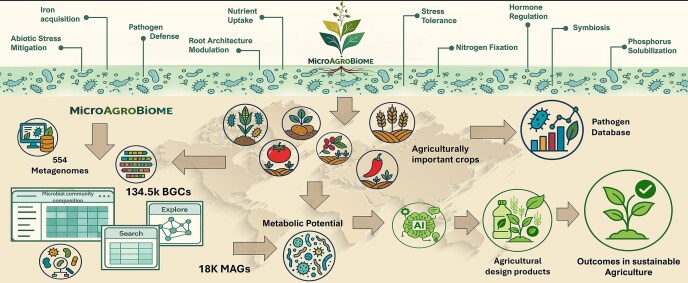

## Introduction

Plants host diverse microbial communities that contribute significantly to their growth, stress resilience, and defense against pathogens [1, 2]. While individual microbes may exhibit beneficial traits, these traits often emerge from community-level interactions that cannot be easily predicted [3]. Soil health depends on several intertwined factors: microbial diversity, host–pathogen genetics, and environmental conditions [4].

Microbial communities, including bacteria, fungi, archaea, protists, and even viruses, can provide crops with direct benefits such as nutrient solubilization, nitrogen fixation, and phytohormone production [5], as well as indirect benefits that act through host-mediated processes, including pathogen suppression, induction of systemic resistance, and enhanced stress tolerance [6]. These interactions can ultimately shape plant phenotypes and adaptability [7].

Certain microbial lineages, referred to as “core microbiota,” consistently associate with specific hosts across environments [8]. The distribution of differential microorganisms, including those in the core of each crop, can help establish a baseline through which individual crop microbiomes can be studied.

Current microbiome databases are largely general-purpose [9–13] and offer limited focus on crop-associated environments (Table 1). To fill this gap, we developed MicroAgroBiome, a dedicated platform that integrates curated rhizosphere metagenomes from different whole metagenome studies with standardized annotations and metagenome-assembled genomes to support microbiome-informed agricultural research.

**Table 1.** Shotgun microbiome repositories compendium

Platform	Crop–specific	MAGs/BGCs	AMR data	Co-occurrence/network tools	Reference
MicroAgroBiome	Yes	Yes	Yes	Yes	This work
AgMicrobiomeBase (UK)	Yes (UK crops)	Yes	(limited)	No	[12]
MGnify	No	Yes	Yes	Yes	[11]
IMG/M	No	Yes	Yes	Yes	[9]
ARM database	No	Yes	No	No	[13]
PLaBAse	No	Yes	(limited)	No	[10]

To contextualize microbial diversity within agricultural systems, MicroAgroBiome focuses on three features: microbial biosynthetic potential, pathogen presence, and abundance tables for ecological networks. Biosynthetic potential highlights beneficial microbial functions such as phytohormone production, siderophore activity, and antimicrobial compound biosynthesis, while pathogen detection is essential given its role as a major driver of crop yield loss. Ecological networks capture the complexity of microbial interactions beyond single taxa, identifying keystone species that can guide the design of beneficial synthetic communities. Although linking microbiomes to crop-specific traits is a central goal of MicroAgroBiome, in this first release, the only crop trait currently integrated is the cropping system (monoculture versus mixed). The platform, however, has been designed to incorporate additional agronomic traits such as yield, fruit quality, or plant health indicators as standardized metadata becomes available, paving the way for future predictive models connecting microbiomes with agricultural outcomes.

## Materials and methods

### Metagenome selection and curation

Metagenomes were selected from public repositories according to the following criteria: completeness and consistency of associated metadata, use of shotgun sequencing methodology, inclusion of a wide range of crop-associated samples (28 different food crops), and broad geographic coverage encompassing multiple regions, countries, and continents. To ensure data reliability and traceability, we prioritized studies published in peer-reviewed journals that provide enough metadata for each sample. Based on these criteria, we compiled a curated list of accession IDs and an accompanying metadata table.

### Bioinformatic workflow

Data processing was performed through a fully automated, Makefile-based workflow that sequentially handled each analysis step, including raw data retrieval, quality control, assembly, taxonomic classification, functional annotation, and targeted read extraction. All scripts are executed in a conda environment inside a Docker container. Code is available at the GitHub repository: https://github.com/aapashkov/generic-metagenomics/tree/rhizosphere. This pipeline may be locally installed and applied to annotate metagenomes of the users regardless of their origin.

### Taxonomic and functional annotation

Taxonomic classification was conducted using Kraken v2.1 [14] with a prebuilt database released on 09 October 2023 (https://benlangmead.github.io/aws-indexes/k2). Functional annotation at the read level was performed with MiFaser v1.61 [15] using the GS-21-all reference database.

### Metagenome-assembled genomes and biosynthetic gene clusters

Before assembly, raw reads were trimmed for quality and adapter removal using TrimGalore v0.6.10 [16], with a minimum read length threshold of 40 base pairs. Assemblies were generated with MEGAHIT v1.2.9 [17], and binning was performed using MaxBin v2.2.7 [18] under default settings. Metagenome-assembled genome (MAG) quality assessment was conducted with the lineage_wf pipeline of CheckM v1.2.2 [19], with reference database as of 16 January 2015. Taxonomic assignment of MAGs was carried out with the classify_wf pipeline of GTDB-Tk v2.4.0 [20], using the GTDB r220 reference database. Biosynthetic gene cluster (BGC) identification was performed using antiSMASH v6.1.1 [21] in Bacteria mode, with Prodigal v2.6.3 [22] in metagenome mode as the gene-calling tool.

### Antimicrobial resistance prediction and plant pathogen database

Antimicrobial resistance (AMR) markers—including genes and SNPs—were predicted using RGI v6.0.3 [23], configured to detect loose, nudged, and low-quality hits. Sequence alignment was performed with DIAMOND v0.8.36 [24] against the CARD v3.2.8 database [23]. The current version of our plant pathogen database is based on the list of Common Names of Plant Diseases of the American Phytopathological Society.

### Co-occurrence network analysis

Co-occurrence networks were computed from BIOM-formatted abundance tables with in-house Python scripts. For each pair of species, two metrics were calculated: Bray–Curtis dissimilarity and Spearman correlation. Species were considered to co-occur if Bray–Curtis dissimilarity was <0.3 and Spearman correlation was >0.7 with *P* < .05. Correlations were classified as positive (abundance of one species increases with the other) or negative (abundance of one species decreases with the other). To strengthen inference, both bootstrap and permutation tests were applied by creating 1000 simulations for each mode. Then, the *P*-values are calculated and reported for all scores. For targeted analyses, a “single taxon mode” is available, which compares a specific taxon (by TaxID) against all others in the dataset. This approach significantly reduces computation time compared to full network reconstruction.

### Site architecture

The platform was coded in Python using the Flask library. The database is stored in SQL, while the metadata for the samples is stored in a CSV file. For the co-occurrence networks, a work queue was implemented to support running times on the scale of hours, depending on the size of the BIOM file. By default, this process will use all the cores available in the system except one.

## Results

### MicroAgroBiome: a specialized platform

MicroAgroBiome fills a gap in agricultural microbiome research by offering a tailored platform for the study of crop-related microbiomes (Table 1). Unlike general metagenomic databases [9–13, 25], MicroAgroBiome contextualizes microbial diversity by linking it with crop-specific traits, pathogen presence, biosynthetic potential, and ecological networks [26, 27]. With an intuitive web interface, it integrates taxonomic and functional data to support discovery and application, enabling innovations such as insights into the composition of microbial synthetic communities for improving crop resilience and yield [28, 29]. MicroAgroBiome’s mission is to catalyze interdisciplinary collaboration and advance microbiome-informed strategies for sustainable agriculture and food security.

### Database overview

**Figure 1.**
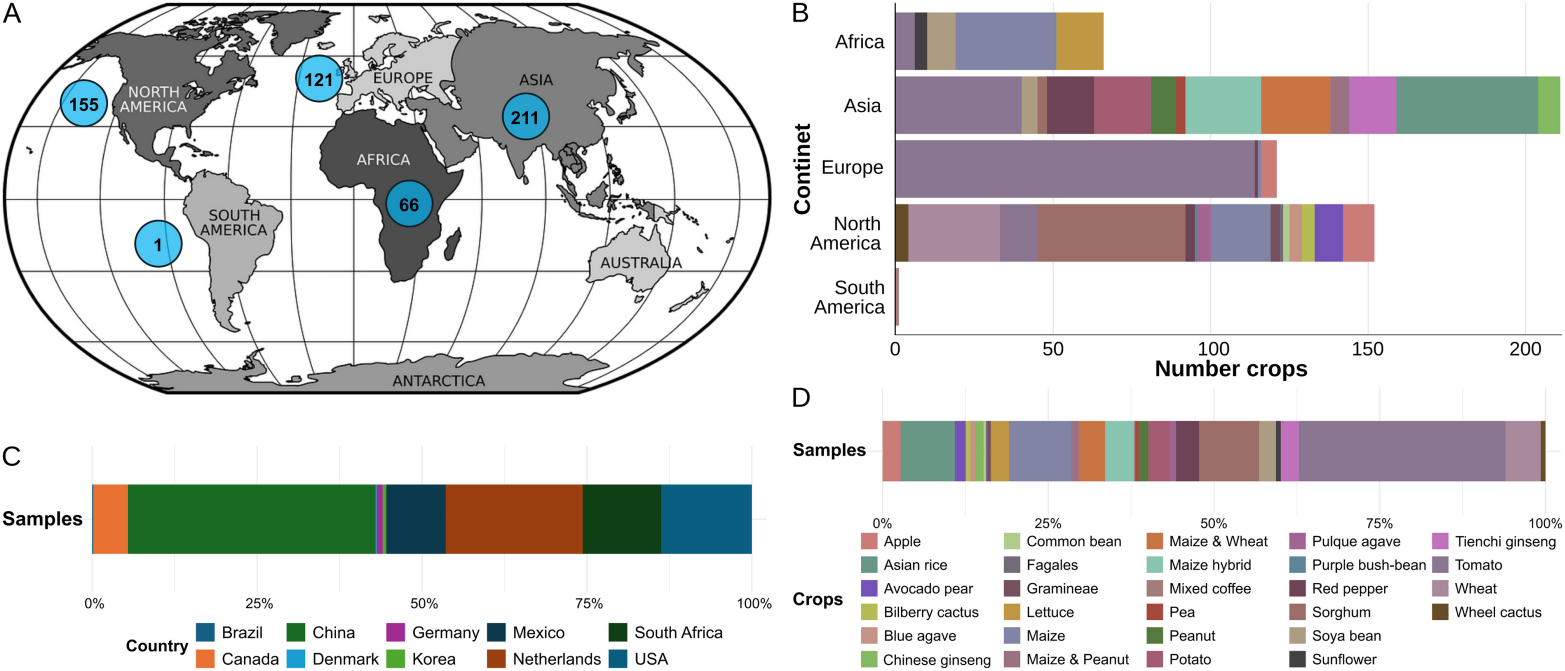
Geographic and crop-based distribution of samples in the MicroAgroBiome database. (**A**) Global origin of the samples. (**B**) Sample distribution by continent; crop type is indicated by the color in the legend at the bottom in the right side of the panel. (**C**) Distribution of all metagenomes included in the MicroAgroBiome platform according to their country of origin and sorted alphabetically. A total of 10 countries are represented. (**D**) Distribution of all metagenomes included in the MicroAgroBiome platform according to their crop of origin. A total of 28 crop species are represented in alphabetical order. Stacked bars in panels (C) and (D) represent the proportion of metagenomes, summing to 100%.

MicroAgroBiome compiles standardized metagenomic data from 554 samples across 28 food crops and soil samples, collected on four continents—most notably in China and the Netherlands (Fig. 1A and B). Tomato and maize are the most represented crops, comprising ∼41% of samples (Fig. 1C and D). Each sample was processed through standardized pipelines to ensure consistent taxonomic and functional annotations.

From this global dataset, the platform has reconstructed over 20 289 bins, 739 of which meet the quality standards for being considered MAGs with >90% completeness and <10% contamination. For each bin, MicroAgroBiome provides functional annotation, BGC mining, and antibiotic resistance analysis. In total, 139 900 BGCs were annotated, with 6088 of them belonging to high-quality MAGs. This foundational resource enables a detailed study of microbial community composition and metabolic potential across various crops and environments.

**Figure 2.**
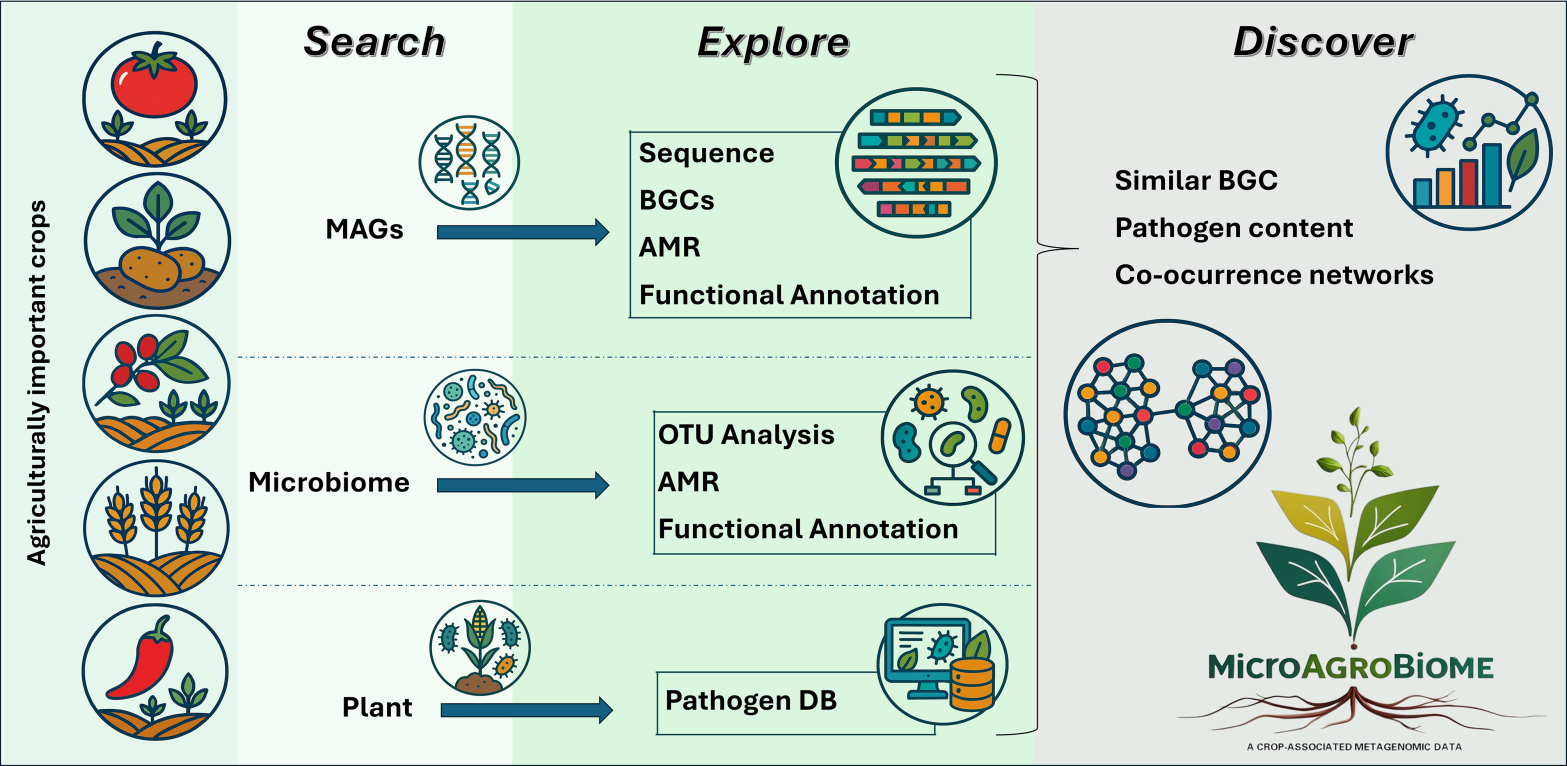
MicroAgroBiome site map. MicroAgroBiome integrates rhizosphere metagenomes from various agriculturally important crops, processing them along three main analytical approaches. First, the metagenomic data are used to reconstruct MAGs, which are then analyzed to identify BGCs, AMR genes, and functional annotations. Second, data are used to generate comprehensive microbiome profiles, including OTU analysis, community-level functional annotation, and identification of AMR genes. Third, the metagenomes contribute to the development of a curated pathogen database, which aims to identify potential plant pathogens present in the rhizosphere. Each of these analyses is available on the platform, both as individual tools and combined. Additionally, MicroAgroBiome offers tools to explore the data through co-occurrence network analysis and comparative assessments of BGCs and pathogen content, providing a flexible framework for understanding the composition, function, and potential impact of microbiomes associated with crops.

MicroAgroBiome’s interface is divided into three main modules: Search, Explore, and Discover, each offering tools to analyze microbial diversity, detect core taxa and pathogens, visualize co-occurrence networks, and assess biosynthetic capacity. Datasets include information on antimicrobial resistance, phytopathogen prevalence, and crop-specific microbiota (Fig. 2). All data (OTU tables, functional annotations, MAGs, and code) are fully downloadable and open access, ensuring transparency and reproducibility.

### MicroAgroBiome navigation

The MicroAgroBiome platform offers a streamlined and intuitive interface. Upon entering the portal, users are introduced to the project’s objectives and provided with direct access to its main features. At the core of the platform is the Search functionality, which allows users to browse data by sample type, plant species, or run ID. This feature is supported by a visual Site Map that guides users through the available datasets and analytical tools, helping them navigate the platform with ease. For those interested in conducting their own analyses, the Downloads section provides access to key datasets in commonly used formats, including .biom, .tsv, and .csv. These files contain abundance data, comparative profiles, and metadata, enabling researchers to perform customized analyses offline or integrate the data into their own pipelines. The platform also offers a “Process Your Sample” section, where users can upload their own microbiome data in either Kraken.report or .biom formats. After assigning a unique run name and selecting the appropriate sample type, users simply upload their file and initiate processing. Upon completion, the user is automatically directed to the Check Results page, where the status and output of the analysis are displayed. Results vary by input type: Kraken files yield detailed taxonomic classification tables and Sankey-style visualizations, while BIOM files provide abundance tables and co-occurrence networks, both of which are available for download in .csv format.

**Figure 3.**
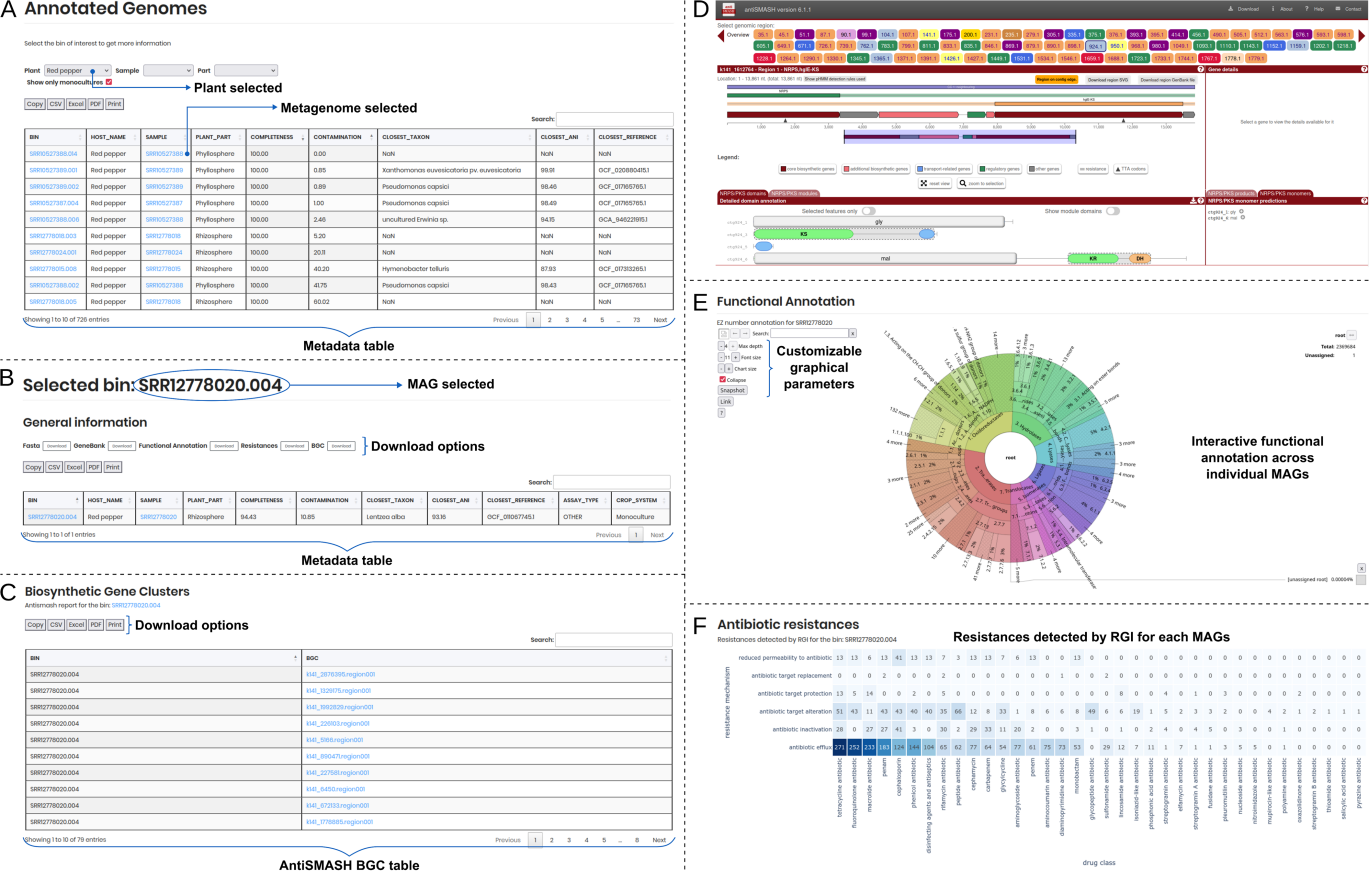
MicroAgroBiome Navigation. (**A**) MicroAgroBiome provides access to MAGs, allowing users to explore individual microbial genomes recovered from crop rhizospheres. (**B**) MAGs can be examined in detail to assess taxonomic identity, genomic structure, and functional potential. (**C**, **D**) A key feature is the integration of BGCs, which supports investigations into secondary metabolite production and the discovery of novel natural products. (**E**) Functional annotation of each MAG can be explored in an interactive graphic with customizable parameters. (**F**) In addition, each genome includes AMR profiles, offering insight into the spread and prevalence of resistance genes in agricultural microbiomes, an increasingly important factor for plant and environmental health.

Importantly, MicroAgroBiome provides access to MAGs, enabling detailed exploration of microbial genomes from crop rhizospheres. Users can assess taxonomic identity, genomic structure, functional potential, and BGCs, supporting natural product discovery. Each genome also includes AMR profiles, offering insights into resistance gene prevalence in agricultural environments (Fig. 3A–F). To further support users, the platform includes example analyses such as a *Clavibacter* case study. This example demonstrates how to construct co-occurrence networks using genus-level OTU tables derived from chili, corn, and tomato samples. It illustrates the use of Bray-Curtis dissimilarity and Spearman correlation to detect associations between Clavibacter and other microbial genera, supported by network visualizations and Venn diagrams that reveal host-specific microbial patterns. For more detailed information about the navigation of the platform, you can access to https://ccm-bioinfo.github.io/microagrobiome.

### Annotated genomes contain MAGs, BGCs, and AMR annotations

For each crop, users can visualize MAGs and bins (Fig. 3A) and download them in multiple formats, such as FASTA and GenBank (Fig. 3B). Each of the 20 289 bins is annotated with its estimated completeness and contamination, as well as its closest taxon in the Genome Taxonomy Database [20], determined using average nucleotide identity and cross-referenced with the NCBI Taxonomy database [30]. On click, every bin displays BGC predictions (Fig. 3C and D), hierarchical functional annotation (Fig. 3E), and AMR predicted markers clustered by resistance mechanism (Fig. 3F).

### Microbiome: taxonomic assignment and core microbiota

**Figure 4.**
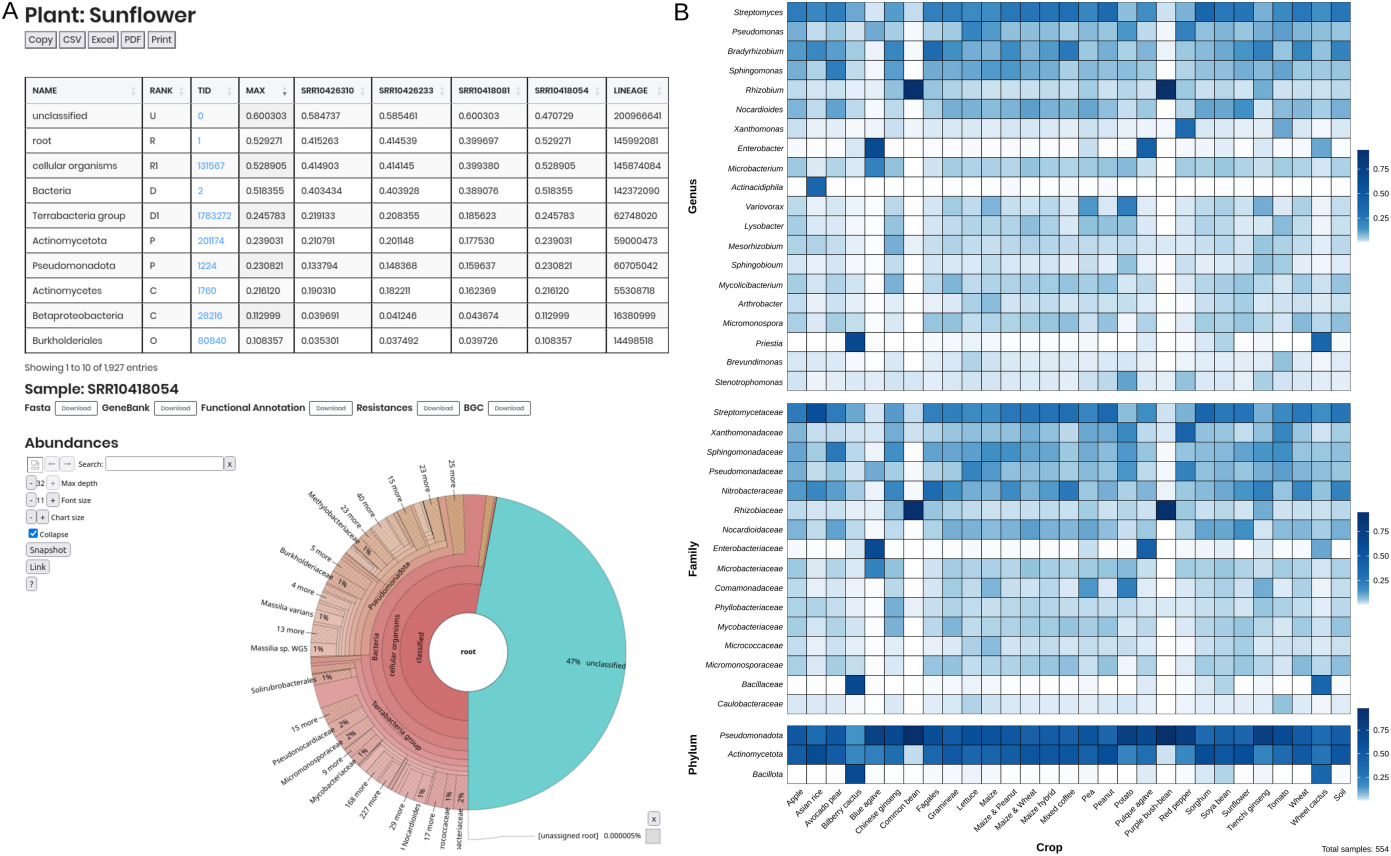
Interactive abundance visualization and downstream analyses. (**A**) Taxonomic classification results displayed through an interactive, user-friendly interface from the MicroAgroBiome platform. The panel shows a comparative abundance table alongside a Krona-style hierarchical visualization, allowing exploration of microbial composition from metagenomic datasets at different taxonomic levels. (**B**) Heatmaps showing the relative abundance of the top 20 most abundant bacterial genera (excluding the genus Human) across different crops. For these top genera, the most abundant families and, subsequently, the most abundant phyla are displayed. These visualizations highlight patterns of microbial dominance and potential crop-specific taxonomic associations.

The MicroAgroBiome microbiome tab displays metagenomes, sorted by crop, showing comparative taxonomic distribution. Upon clicking, abundance profiles are displayed individually for the selected sample in an interactive Krona graph [31] (Fig. 4A). These tools support a wide array of downstream analyses: identifying core microbiomes, calculating alpha and beta diversity, assessing dominant taxa, evaluating metabolic capabilities, and flagging potential pathogens. This facilitates a deep understanding of the ecological and functional aspects of crop-associated microbial communities. As an example, we show the top taxa in different crops, where according to another soil atlas [32], Pseudomonadota and Actinomycota are the most abundant phyla in crops (Fig. 4B).

### Plants

This tab shows a plant catalog that comprises several pathogens accompanied by their tax ID and the diseases they cause.

### Downstream analysis

We illustrate three downstream analysis workflows: AI powered microbiome classification, profiling crop-specific BGC content, and mapping *Clavibacter* interaction networks.

#### AI powered microbiome classification

Artificial intelligence (AI) is revolutionizing microbiome research by uncovering complex patterns in metagenomic datasets, such as those in MicroAgroBiome. Machine learning links microbial taxa, gene functions, antimicrobial resistance, and environmental contexts, revealing adaptive traits and ecological niches [33–35]. AI also supports the rational design of synthetic microbial consortia aimed at improving nutrient uptake, enhancing disease resistance, and restoring degraded soils. Understanding the relevant variables enables “biological prescriptions” for agricultural systems [36], analogous to microbiome transplants in medicine. This approach, known as agricultural microbiome engineering, offers a sustainable and precision-driven method for crop management. MicroAgroBiome is poised to become a resource for this AI-driven future, as its growing dataset offers the scale, data standardization, and resolution needed for effective model training.

**Figure 5.**
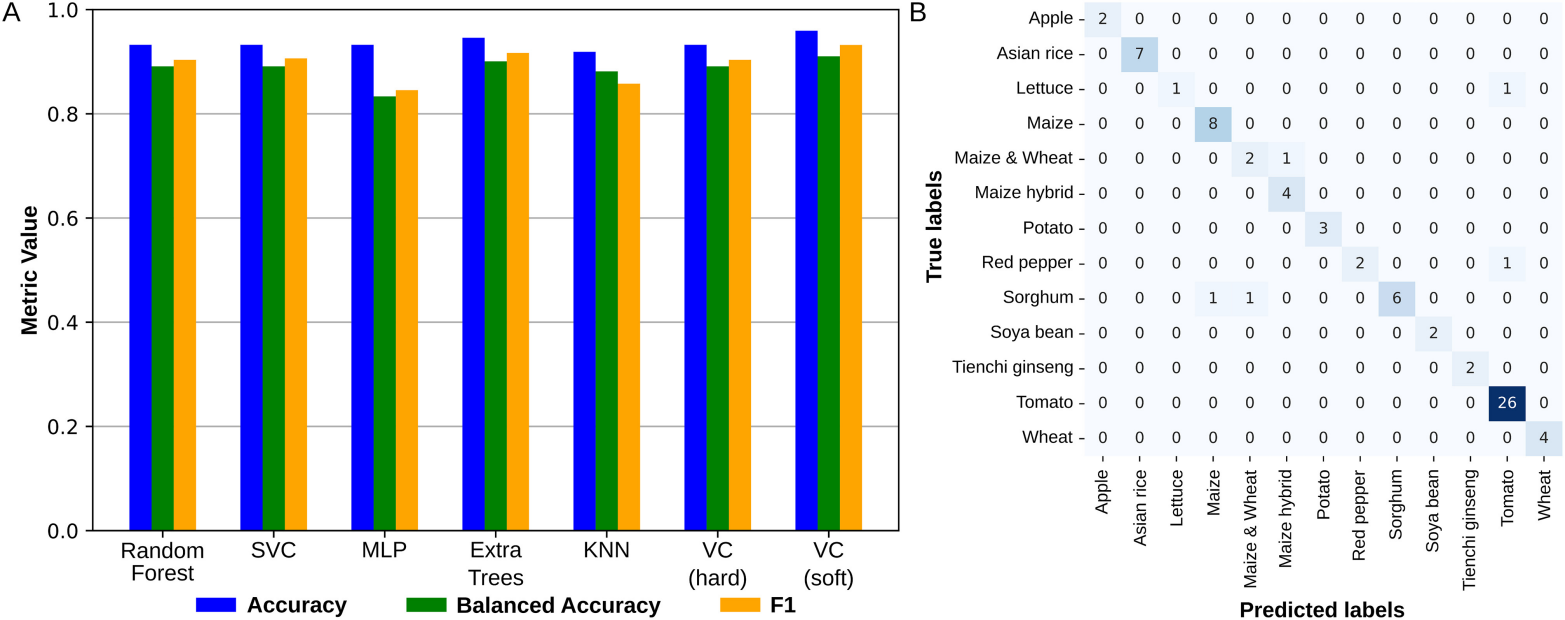
Performance evaluation of classification models. (**A**) Comparison of classification performance across models based on accuracy, balanced accuracy, and F1 score. Bars represent the mean values obtained from cross-validation. The classification models yielded accuracies ranging from 0.945 to 0.986, balanced accuracies from 0.846 to 0.961, and F1 scores from 0.834 to 0.973. (**B**) Confusion matrix of the SVC model showing classification performance on the validation data.

Some crops may have a particular microbial community that allows them to be distinguished from others. Here, we trained models to predict the origin of a crop from the available microbial distribution. For model training, we included only those crops with >10 recorded observations to ensure sufficient data representation. Algorithms follow the code previously used to classify microbiomes from different cities [33] and use a five-fold cross-validation strategy with 4:1 training to validation sets. Metagenomes in MicroAgroBiome were accurately classified by crop with supervised learning (Fig. 5A). Among classification algorithms, the voting classifiers (VC), both hard and soft voting, and the support vector classifier (SVC) (Fig. 5B) achieved the highest accuracy and F1 scores, with balanced accuracies above 0.96. Code for crop prediction uses Python 3.9.19, with libraries scikit-learn 1.5.0, pandas 2.2.1, seaborn 0.13.2, and matplotlib 3.9.0.

#### Crop-specific BGC content

**Figure 6.**
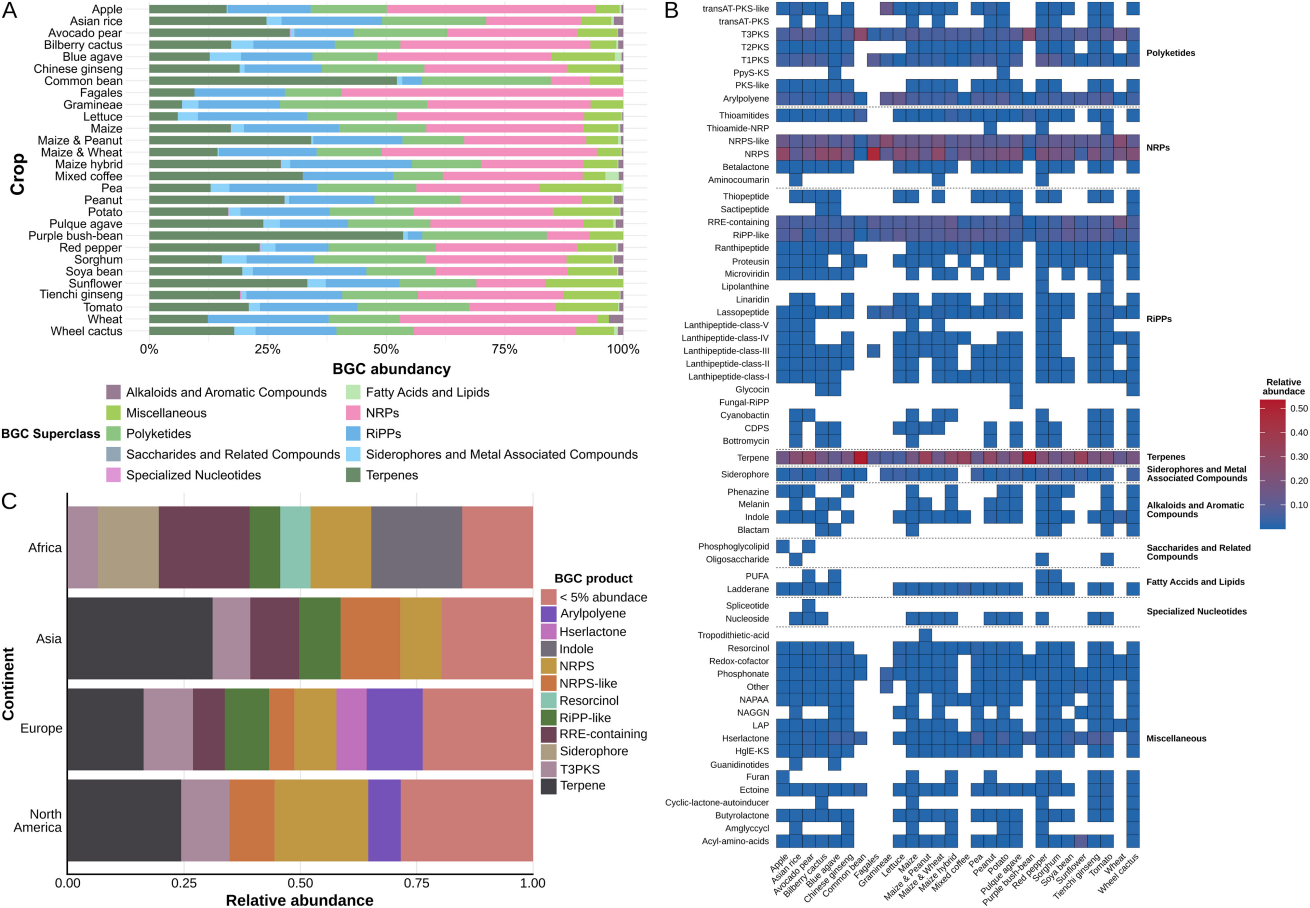
Classification and distribution of BGCs recovered from MAGs reconstructed using rhizosphere metagenomic datasets. (**A**) Worldwide BGC distribution organized by crop and superclass. (**B**) Worldwide BGC heatmap of best-supported crops organized by class and superclass. (**C**) Tomato BGC class abundance per region.

The platform’s MAG and BGC resources are central to MicroAgroBiome’s value. With 139 900 BGCs across 28 crop species and soil, users can analyze secondary metabolite diversity globally or crop-specifically (Figs. 6A–C). These clusters are categorized by class and superclass to aid in the study of metabolic potential and ecological function. For microbial natural product discovery, this is a rich resource for reconstructing biosynthetic pathways and identifying novel enzymes or unusual BGC architectures. These components (often referred to as “biochemical dark matter”) represent untapped metabolic diversity with promising bioactivities. Advances in computational tools now enable the systematic mining of such data using evolutionary frameworks [37]. For example, tools such as EvoMining and CORASON [38] trace biosynthetic gene evolution, helping uncover novel pathways and molecules. This strategy—evolutionary genome mining—is reshaping functional genomics and natural product discovery [39].

#### Mapping Clavibacter interaction networks

*Clavibacter* is a relevant plant pathogen; some species of this genus infect tomato, corn, and chili. As an example of the use of co-occurrence networks, we use crop-associated microbiomes to find microorganisms whose presence is associated with *Clavibacter*. We use 18 microbiome samples of tomato, 34 samples of corn, and 13 samples of chili. We found several interactions with Actinomycetes, which is the *Clavibacter* class; most of these are positive correlations. At the genus level, we found two genera consistently correlated with *Clavibacter: Bifidobacterium* and *Curtobacterium*. The presence of *Curtobacterium* may be explained by it being an opportunistic infection after *Clavibacter* infection in the plant. Further analysis and figure are shown in Supplementary data.

## Discussion

MicroAgroBiome is a dedicated platform designed to advance our understanding of rhizosphere microbiomes in agriculturally important crops. Unlike general-purpose metagenomic repositories, it offers a crop-centered, standardized framework that enables the exploration of microbial diversity, ecological interactions, and functional traits directly relevant to plant health and productivity.

Currently, the platform hosts 554 standardized metagenomes from 28 food crops, collected across 4 continents. While this dataset already enables robust comparative and functional analyses, MicroAgroBiome is actively expanding and is designed to become the largest global repository of crop-associated metagenomic data. By continuously incorporating new samples, it will enhance its capacity to represent geographic, environmental, and crop-specific diversity. In addition to core taxonomic and functional profiling, the platform supports co-occurrence network analysis, pathogen detection, and antimicrobial resistance screening—all through an intuitive interface that facilitates data access, visualization, and custom downstream analysis.

MicroAgroBiome goes beyond cataloging microbial diversity; it enables actionable insights. Its integration of taxonomic, functional, and biosynthetic data supports targeted research in microbial ecology, plant–microbe interactions, and soil health. While MicroAgroBiome does not currently include physicochemical soil parameters, it provides a microbiological perspective by offering a functional blueprint of the soil microbiome. These data serve as a foundation for designing synthetic microbial consortia with restorative capabilities, which can later be complemented with abiotic soil information. With artificial intelligence, the platform could identify beneficial microbial consortia, predict their impact under diverse scenarios, and ultimately enable tailored biological interventions.

Looking ahead, future metagenomic studies of rhizosphere microbiomes should expand beyond the current focus on annual crops to include perennial species, which are central to agricultural sustainability yet remain underrepresented. Incorporating longitudinal sampling across plant developmental stages and seasons will help capture temporal dynamics of microbial communities. At the same time, increasing coverage from underexplored regions such as Latin America and Africa will improve global representation and reduce geographic bias. The integration of multi-omics approaches—including metatranscriptomics, metabolomics, phenotyping, and long-read sequencing—together with abiotic variables such as soil type, nutrient status, and temperature, will be critical to link microbial diversity with ecological functions and agronomic traits. Together, these directions will provide the foundation for predictive models and data-driven strategies to harness rhizosphere microbiomes for sustainable agriculture.

In summary, MicroAgroBiome is not just a database; it is a research and innovation platform that connects biodiversity, functional genomics, and computational tools to accelerate the development of sustainable, microbiome-based agricultural strategies. Its depth, interoperability, and focus on practical application position it as a foundational resource for the future of crop microbiome science and regenerative farming.

## Supplementary Material

gkaf1083_Supplemental_File

## Data Availability

All data in MicroArgobiome are public data, and original NCBI Ids are shown in the database. Code is available at GitHub (https://github.com/aapashkov/generic-metagenomics/tree/rhizosphere) and Zenodo (https://doi.org/10.5281/zenodo.17246890), while documentation is located at https://ccm-bioinfo.github.io/microagrobiome.

## Notes

Present address: Centro de Investigación y de Estudios Avanzados del Instituto Politécnico Nacional, Unidad Irapuato, Libramiento Norte León Km 9.6, Irapuato, Guanajuato, 36821, México
