# Supplementary material for: MicroAgroBiome: a toolkit for exploring specialized metabolism and ecological interactions in rhizosphere microbiomes of cultivated crops
Source: Nucleic Acids Res. 2025 Nov 17;54(D1):D1743–52. doi: 10.1093/nar/gkaf1083 (PMC12807682; doi:10.1093/nar/gkaf1083)
Supplement: gkaf1083_Supplemental_File [file gkaf1083_supplemental_file.pdf]

Supplementary material AgroMicroBiome

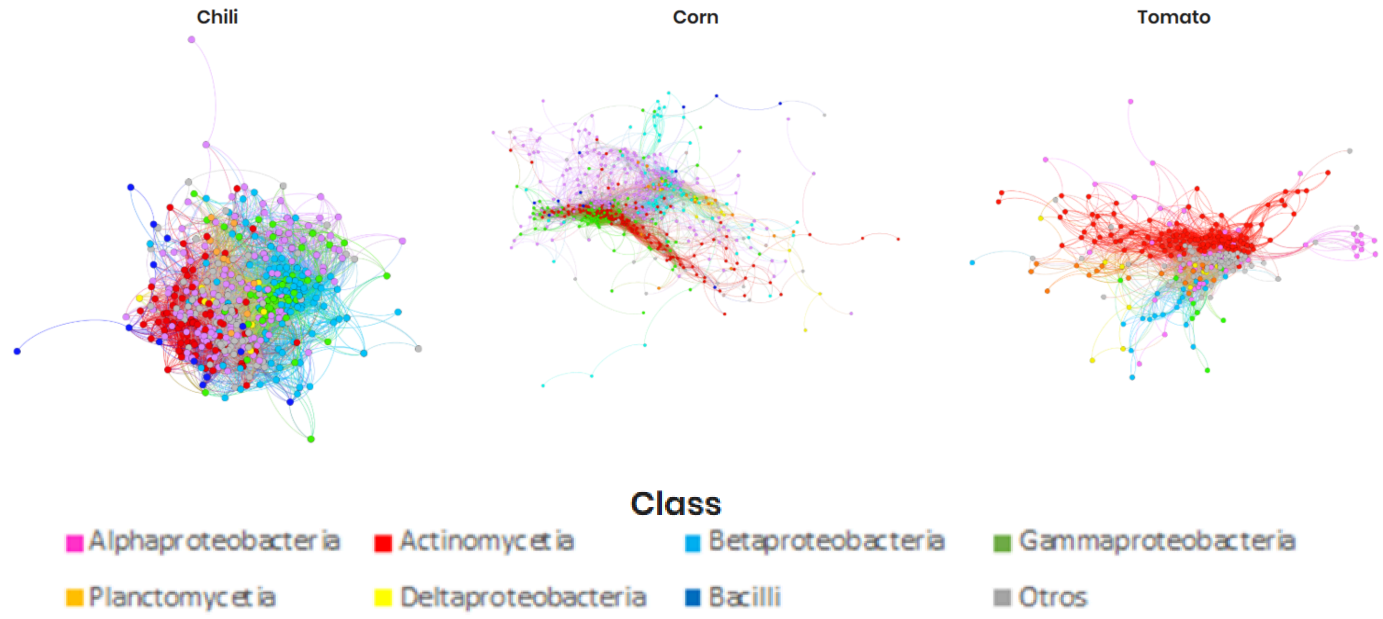

Fig S1 Co-occurrence networks for corn, chili and tomato microbiomes.

Co-occurrence networks for corn, chili and tomato. We used 18 samples for tomato, 34 for corn, and 13 for chili. Abundance counts were calculated with Kraken2 (Wood 2019) and aggregated to the class level. The co-occurrence networks were calculated using the Bray-Curtis dissimilarity measure and Spearman's weighting (Lima 2015). A link is established between two classes if their dissimilarity is less than 0.3 and their weight is higher than 0.77. Visualization and community detection were generated with Gephi (Bastian 2009).

Several classes associated with Actinomycetia (Clavibacter class) were found in different cultures; most of them were found to be positive correlations. At the genus level, two genera correlated with Clavibacter were found: Bifidobacterium and Curtobacterium on all plants. Curtobacterium is an opportunistic infection after Clavibacter. Bifidobacterium correlation needs to be studied further. In Chile we found six negatively correlated genera: Porphyrobacter, Achromobacter xylosoxidans, Brucella, Sphingobacteriaceae, Thermomonas and Achromobacter.
